# Diversifying Selection in Plant Breeding

**DOI:** 10.1371/journal.pbio.0020347

**Published:** 2004-10-12

**Authors:** Susan McCouch

## Abstract

Natural variation rather than genetic modification - is this the way to achieve global food security?

*“Some qualities nature carefully fixes and transmits, but some, and those the finer, she exhales with the breath of the individual as too costly to perpetuate. But I notice also that they may become fixed and permanent in any stock, by painting and repainting them on every individual, until at last nature adopts them and bakes them into her porcelain”—Ralph Waldo Emerson*

The history of domesticated plant form and function evolves along a two-tiered track that doubles back on itself, offering panoramic vistas of natural forces intertwined with the creative force of human endeavor (Figure 1). For approximately 10,000 years, human beings have modified the traits of plants and animals, giving rise to hundreds of thousands of domesticated breeds that today form the foundation of the world's food supply. Modern breeds are descendents of the wild species from which they were derived. The process of domestication dramatically changed the performance and genetic architecture of the ancestral species through the process of hybridization and selection as originally described by Charles Darwin (1859).

**Figure 1:**
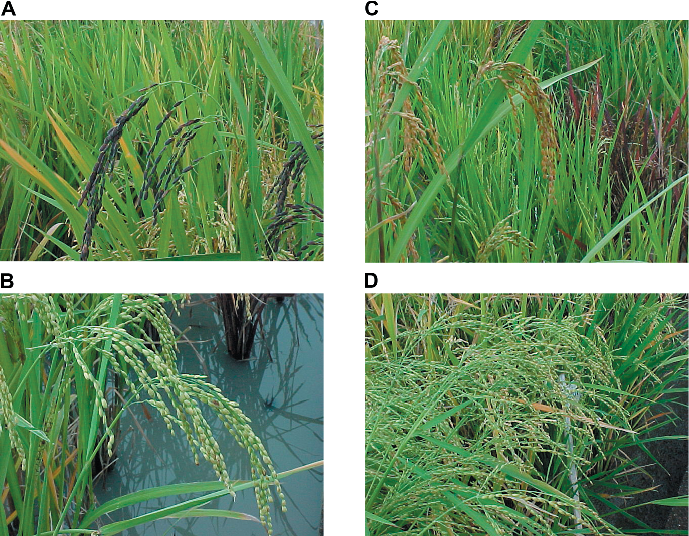
The Diversity of Ancestral Rices (A) Long, thin-grained rice with purple hull. (B) Round-grained rice with white hull. (C) Panicles of golden-hulled rice (foreground) and purple-leafed rice (background). (D) Tall, weedy rice with pale leaves and silver hulls.

Despite the low yields and poor eating quality of most wild ancestors and primitive crop varieties, these ancient sources of genetic variation continue to provide the basic building blocks from which all modern varieties are constructed. Breeders have discovered that genes hidden in these low-yielding ancestors can enhance the performance of some of the world's most productive crop varieties. In this essay, I will provide some historical context for the paper by Gur and Zamir in this issue of *PLoS Biology* (Gur and Zamir 2004). I will discuss how “smart breeding” recycles “old genes” to develop highly productive, stress-resistant modern varieties and why this approach is particularly attractive to increase food security in regions of the world with high concentrations of genetic diversity.

The job of the plant breeder is to create an improved variety. This may be accomplished simply by selecting a superior individual from among a range of existing possibilities, or it may require that a breeder know how to efficiently swap or replace parts, recombine components, and rebuild a biological system that will be capable of growing vigorously and productively in the context of an agricultural environment. How the breeding is done and what goals are achieved is largely a matter of biological feasibility, consumer demand, and production economics. What is clear is that the surest way to succeed in a reasonable amount of time is to have access to a large and diverse pool of genetic variation.

The process of plant breeding is theoretically simple, but its power resides in the fact that it creates novelty. A breeder generally selects two individuals for crossing, each of which has specific traits or characteristics of interest. The cross provides the mechanism by which genes are exchanged between the parents so that a wide array of diverse individuals is observed in the progeny of future generations. From a breeding perspective, this provides the basis for selection so that individuals containing the best features of both parents can be identified and further bred. By selecting parents that are genetically similar, a breeder restricts the amount of variation that will be evaluated in the offspring. On the other hand, by crossing genetically divergent parents, the range of phenotypic variation will be much more extensive and can even be surprising, with many individuals presenting phenotypes that would not be expected based on the attributes of the parents. Thus, if a breeder is interested in innovation and wants to generate maximum variation from which to make selections, wide crosses are the most productive.

Not all genetic variation is created equal. When Darwin first introduced the concept of evolution (Darwin 1859), he challenged the prevailing view that species were fixed entities with a single, invariable genetic identity. The concept of natural selection presupposed that species were comprised of genetically variable individuals such that selection could act on them. The genetic variants differ in the alleles (versions of genes) they carry. Alleles that are deleterious in terms of the survival and reproduction of the organism will eventually be eliminated while alleles that are favorable or neutral will be perpetuated in the population. Recombination in natural populations allows alleles that may be deleterious in one genetic background to be reassessed in a different genetic context. Over time, the alleles that are transmitted at high frequency across generations represent those with a substantial likelihood of contributing positively to an organism's long-term viability in a variable environment. For this reason, natural variation is a much more valuable and informative reservoir of genes for the purposes of plant improvement than would be an equivalent number of induced mutations generated in a laboratory.

## Domestication—The Winnowing of Natural Genetic Variation

Cultivars (domesticated varieties) have been selected by humans in the last 10,000 years and inevitably represent a subset of the variation found in their wild ancestors. Cultivars are recognizable because they manifest characteristics that are associated with domestication in plants. Unusual or extreme phenotypes, such as large fruit or seed size, intense color, sweet flavor, or pleasing aroma are often selected by humans and maintained in their cultivars for aesthetic reasons, while synchronous ripening or inhibition of seed shattering (a dispersal mechanism) are selected to facilitate harvest. These phenotypes may occur in nature but they will frequently be eliminated by natural selection before they are fixed in a population. Because of human selection, cultivars may exemplify a range of exaggerated phenotypic attributes that give them the appearance of being, on the whole, more diverse than some of the wild populations from which they were derived, but in truth, domestication usually represents a kind of genetic bottleneck. Furthermore, cultivars are grown in agricultural environments that are generally more uniform than the environments in which wild species grow, and this tends to further narrow the gene pool. Thus, while cultivars may embody a high degree of obvious phenotypic variation, this may not always be a good predictor of the extent of their genetic variation.

**Figure 2:**
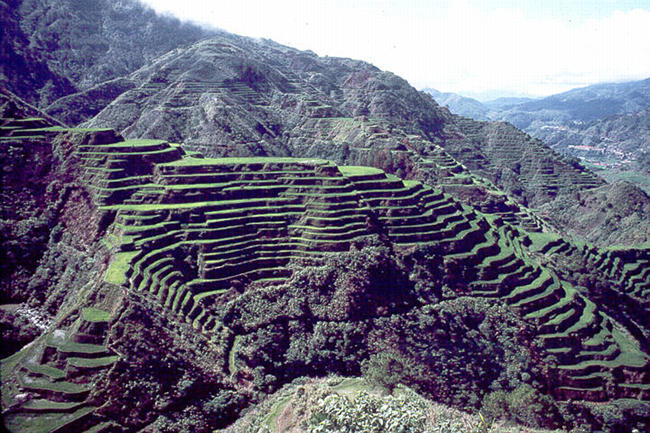
Banaue Rice Terraces in the Philippines Where Traditional Landraces Have Been Grown for Thousands of Years

The landrace varieties are the earliest form of cultivar and represent the first step in the domestication process. Landraces are highly heterogeneous, having been selected for subsistence agricultural environments where low, but stable yields were important and natural environmental fluctuation required a broad genetic base (Figure 2). Landraces are closely related to the wild ancestors and embody a great deal more genetic variation than do modern, high-yielding varieties that are selected for optimal performance within a narrow range of highly managed environmental conditions. The value of both the wild species and the early landrace varieties in the context of modern plant breeding is that they provide a broad representation of the natural variation that is present in the species as a whole. The fact that natural selection has acted on such populations over the course of evolution makes them particularly valuable as materials for breeders. The value added by imposing a low intensity of human selection on the early landraces resides in the fact that some of these early varieties represent accumulations of alleles that produce phenotypes particularly favorable or attractive to the human eye, nose, palette, or other appetites. It is also noteworthy that some of these rare or unique alleles or allele combinations that were selected by humans might never survive in the wild.

Wild relatives and early landrace varieties have long been recognized as the essential pool of genetic variation that will drive the future of plant improvement (Bessey 1906; Burbank 1914). Early plant collections made by people such as Nikolai Vavilov (1887–1943) or Jack Harlan (1917–1998) inspired the international community to establish long-term collections of plant genetic resources that provide modern plant breeders with the material they need to creatively address the challenges of today (Box 1). Many may question the emphasis on wild and primitive landraces that cannot compete with new, high-yielding varieties in terms of productivity or eating quality, particularly in an age when biotechnology and genetic engineering promise to provide an endless stream of genetic novelty. Indeed, if all forms of novelty were equally valuable, the old varieties would hardly be worth saving. But the security of the world's food supply depends on an exquisite balance between new ideas and the intelligent use of time-tested resources. In 1972, more than a decade before the age of automated sequencing, Jack Harlan commented that, “We are not really much interested in conserving the old varieties as varieties; it is the genes we are concerned about. The old land races can be considered as populations of genes and genetic variability is absolutely essential for further improvement. In fact, variability is absolutely essential to even hold onto what we already have” (Harlan 1972a).

## Combining Breeding with Molecular Genetics

In today's world where automated sequencing and DNA synthesis are mundane activities, it may seem contradictory to be worrying about saving or using “old genes.” Can't new ones be synthesized to order? Can't we modify a plant at will by introducing a new gene or two into an existing variety? Why should we worry about saving populations of historically valuable genes in millions of living plant specimens at great cost to the tax-paying public?

Perhaps it is not the genes themselves we are now in fear of losing. It is the information they encode in all their combinatorial complexity. After all, we are only at the very beginning of the endeavor to understand the way in which a genotype confers a particular set of attributes to a living organism. The subtleties of phenotypic plasticity in the face of a changing environment and the layers of genetic redundancy that characterize biological systems are largely mysterious. We have only just begun to consider the millions and billions of genetic trials and errors that have been evaluated by nature over evolutionary time. We cannot even begin to simulate the selective filters that have provided us with the diversity of form and function in the living world. We do know that living forms of natural diversity are needed to sustain life, and that it would be impossible to replace or recreate that diversity if it were lost at this time.

As plant breeders, we know what to do with living forms of genetic diversity. If we keep our options open and learn to better utilize the reservoirs of natural variation that have been preserved in our gene banks and in the few remaining in situ populations of wild species and landrace varieties, an almost infinite array of novelty can be achieved using traditional, time-proven practices involving crossing and selection of genes that have withstood the test of evolutionary time (Burbank 1914; Hawkes 1958; Rick 1967; Harlan 1975, 1976; Peloquin 1983). By restricting the gene pool, we can readily channel a phenotype into a constrained and predictable outcome. By expanding the gene pool, we can open up many new possibilities for consideration that have not been previously evaluated, would be unlikely to be generated in nature, and would not be readily predicted based on current knowledge.

In crosses between wild and cultivated species of inbreeding plants, alleles that were “left behind” during the domestication process may be reintroduced into the cultivated gene pool. This infusion of “new blood” renews and invigorates modern cultivars in surprising and interesting ways. It is not uncommon for some of the inbred progenies derived from these crosses to perform better than the better parent (Frey et al. 1975; Rick 1976, 1983; Tanksley and McCouch 1997). This phenomenon is known as transgressive variation and results from positive interaction between the genotypes of the parents. Today, plant breeders can analyze populations derived from wide crosses using molecular markers to determine which portions of the chromosomes are associated with the transgressive variation of interest. This makes it possible to dissect a complex phenotype and to determine where individual genes or, more correctly, quantitative trait loci (QTLs) map along the chromosomes. Information about DNA markers linked to QTLs represents a powerful diagnostic tool that enables a breeder to select for specific introgressions of interest, a technique referred to as “marker-assisted selection.”

This approach has proven to be extremely successful in several crop species (tomato [Bernacchi et al. 1998], hybrid rice [Xiao et al. 1998], inbred rice [Thomson et al. 2003], wheat [Huang et al. 2003], barley [Pillen et al. 2003], and pepper [Rao et al. 2003]). In China, two introgressions from a wild relative of rice have been associated with a 30% increase in the yields of the world's highest-yielding hybrid rice (Deng et al. 2004). In tomato, yield increases of greater than 50% resulted from introgressing three independent segments from a wild relative, as reported by Gur and Zamir (2004). The effect of these introgressions on yield was stable in different genetic backgrounds and in both irrigated and drought conditions. This work was facilitated by the availability of a library of chromosome segment substitution lines, called introgression lines when the donor is a wild species, that provided the foundation for exploring the interactions among the independent QTLs. Plant geneticists have long recognized the value of exotic libraries (Brassica [Ramsay et al. 1996; Cermankova et al. 1999], millet [Hash 1999], rice [Sobrizal et al. 1996; Ghesquiere et al. 1997; Ahn et al. 2002], tomato [Monforte and Tanksley 2000; Zamir 2001], wheat [Sears 1956; Pestsova et al. 2002, 2003], and Arabidopsis [Koumproglou et al. 2002]). They represent a permanent genetic resource that greatly facilitates the utilization of wild and exotic germplasm in a breeding program, and they are also an efficient reagent for the discovery and isolation of genes underlying traits of agricultural importance.

## Uncovering the Genes That Underlie Agronomic Traits

Several genes underlying traits of agricultural importance have been cloned using substitution lines derived from interspecific or intersubspecific crosses (Martin et al. 1993; Song et al. 1995; Frary et al. 2000; Yano et al. 2000; Takahashi et al. 2001; Yano 2001), including one of the yield QTLs targeted by Gur and Zamir (Fridman et al. 2000). While the identity of the yield gene conferring the phenotype was not critical to the success of the cultivar development scheme described by Zamir and Gur (2004), there is great curiosity to understand the gene(s) or genes and genetic mechanisms that underlie traits of interest to agriculture. In some cases, knowing the gene or the exact functional nucleotide polymorphism within the gene that determines the phenotype (Bryan et al. 2000; Robin et al. 2002) may dramatically improve the resolution of selection during the breeding process. It also may allow a breeder to make more informed decisions about which germplasm resources to use as parents in a crossing program and which genes within those resources to use in a pyramiding scheme.

As more genes of interest are cloned and their contributions to complex biological systems are better understood, there will be many opportunities for creative synthesis of new varieties. It is likely that some of the opportunities will involve genetic engineering approaches, where new information about genes, gene regulation, and plant responses to the environment may be used in innovative ways to fine-tune existing plant varieties so that they utilize resources more efficiently, provide greater nutritional value, or simply taste better.

## Natural Variation and Food Security

The scientific enterprise has always challenged beliefs about the way the world functions, its origins, and its possibilities. Deeply held beliefs are frequently resistant to the most carefully crafted scientific explanations. When belief systems are unconscious, they may prove particularly resilient to change. Occasionally, science provides an interpretation that fits cleanly into the framework of existing ideas, and then it is heralded with great applause, and often with a sense of relief. When this is not the case, public opinion tends to react fitfully, with many starts and stops. Public opinion has been on a roller coaster recently with respect to transgenic organisms in agriculture. This is in response to what is perceived to be a kind of scientific intrusion into the intimacy of the relationship between humans and their food supply. This relationship is inherently complex, representing a textured fabric of historical, cultural, geographic, economic, biological, and aesthetic concerns. Despite the fact that food is increasingly treated as a commodity in today's global economy, human culture the world over has always recognized that food represents more than a biological remedy for hunger. Food is a force that brings diverse people together, it provides a focal point for human discourse, and it enhances our enjoyment of life. Food also has a spiritual component. Harvesting other living organisms to support human life represents a powerful connection between different spheres of the natural world.

At some level, the idea of using natural genetic variation found in wild species and early landrace varieties to revitalize modern crop varieties is both emotionally appealing and intellectually compelling. As a “smart breeding” strategy, it will facilitate the exploration and utilization of natural genetic variation, expanding the genetic base of our crop plants and providing more flexibility for the future. By using a marker-assisted approach, it will provide a noninvasive road map to expedite the selective introgression of useful traits in the years ahead. Because the approach is primarily useful for self-pollinating species (as opposed to cross-pollinators), variety development can go forward with the expectation that new varieties can be developed and distributed as inbred strains. This will come as very good news to people who are concerned about the infrastructural requirements needed to maintain a hybrid seed industry. Inbred variety seed can be saved from year to year without noticeable loss of vigor. Farmers are free to amplify the varieties and pass seed on to their neighbors if it proves valuable. Plant breeders living in parts of the world where germplasm diversity is highest are in the best position to explore its value. Until now, there have been few opportunities to make use of the wealth of natural diversity that abounds in many countries where people are the poorest and population is growing the fastest. This approach offers a way forward and can help people make good use of locally available resources to enhance the food security of their own nations.

As we consider the implementation of smart breeding efforts in the future, we might ask, who will have access to nature's reserves of genetic diversity? How will knowledge about the patterns that govern the generation and selective elimination of that diversity help guide conservation efforts as well as current and future crop improvement efforts? What are the limits to biological variation? How far can we push those limits, and what will be the consequences of not pushing them? Who will participate in the endeavor? What will the rules of engagement be? What tools can we use to expedite the effort?

What genetic characteristics will help us cope with climate change, global warming, the emergence of new pests and diseases, depleted soils, shortages of fresh water, and increasing levels of water and air pollution? What trace minerals, vitamins, and other metabolites will we need to breed into the crops of the future to fight the causes of hidden hunger, to prevent cancer, or to enhance the immune system? The combinatorial possibilities for crop improvement are almost infinite, as long as we maintain our options. Faced with a clear choice today, it is obvious that enhancing the potential for genetic flexibility in the future is a wise course of action and one we ignore at our peril.

**Box 1. The Pioneers**

*“Moreover, from our wild plants, we may not only obtain new products but new vigor, new hardiness, new adaptive powers, and endless other desirable new qualities for our cultivated plants. All of these things are as immediate in possibilities and consequences as transcontinental railroads were fifty years ago.”—Luther Burbank, 1914*

**Luther Burbank** (1849–1926) was one of America's first and most prolific plant breeders. He was inspired by Charles Darwin's Variation of Animals and Plants under Domestication (Darwin 1883) to explore the potential of creating new varieties of plants by cross-breeding (hybridization) and selection. Over a 50-year period, he developed more than 800 new varieties of fruits, vegetables, flowers, and grasses. One of his earliest creations was the Burbank potato (1871), a variety of baking potato still popular today. When the Plant Patent Act of 1930 was first introduced in Congress, Thomas Edison testified, “This [bill] will, I feel sure, give us many Burbanks.” The bill passed, and Luther Burbank was awarded 16 posthumous patents for asexually reproduced plants (Burbank 1914).

**Nikolai Vavilov** (1887–1943), a Russian geneticist and biologist, was one of the first to explore and actively collect wild relatives and early landrace varieties as sources of genetic variation for the future of agriculture. His botanical collecting expeditions (1916–1940) amassed many thousands of rare and valuable specimens that are preserved in the Vavilov Institute of Plant Industry in St. Petersburg, the world's first seed bank and inspiration for the International Crop Germplasm Collections (http://www.sgrp.cgiar.org/publications.html). Vavilov's concepts in evolutionary genetics, such as the law of homologous series in variation (Vavilov 1922) and the theory of centers of origin of cultivated plants (Vavilov 1926), were major contributions to understanding the distribution of diversity around the world. Vavilov himself died of starvation in a Stalinist prison camp in 1943, victim of a debate about genetics at a time when Trofim Lysenko's theories about the alterability of organisms through directed environmental change proved more compelling to the Soviet leadership than Vavilov's own efforts to demonstrate the genetic value of wild and early landrace diversity.

In the United States, **Jack Harlan** (1917–1998) was also well known for his plant collection expeditions and eloquent expositions about the value of wild relatives and early domesticated forms of crop plants (Harlan 1972b). What particularly sensitized Jack Harlan to the value of these genetic resources was the fact that he lived through a period of revolutionary change in the way agriculture was practiced, watching as the Green Revolution's high-yielding semi-dwarf varieties of wheat and rice replaced the old landrace varieties throughout Asia and Latin America (Harlan 1975). He understood that the new varieties brought massive and immediate increases in grain production that saved millions from starvation. He also understood that displacement of the traditional varieties from their natural environment presented serious challenges that would require renewed efforts to collect, document, evaluate, and conserve plant genetic resources. “For the sake of future generations, we must collect and study wild and weedy relatives of our cultivated plants as well as the domesticated races. These resources stand between us and catastrophic starvation on a scale we cannot imagine” (Harlan 1972b).

**Charlie Rick** (1915–2002) was an avid collector of exotic tomato germplasm. He noted that up until the 1940s, progress in tomato improvement lagged and few major innovations were achieved. The turning point, according to Rick, was the introduction of exotic germplasm. As a cultivated species, tomato had experienced a severe genetic bottleneck that led to extreme attrition of genetic variability compared to the wild species of *Lycopersicon* (Rick and Fobes 1975). Yet, Rick observed that crosses between wild and cultivated species generated a wide array of novel genetic variation in the offspring, despite the fact that routine evaluation of wild and exotic resources often failed to detect the genetic potential of these resources (Rick 1967, 1974). He outlined “pre-breeding” strategies that were designed to uncover positive transgressive variation in backcrossed (inbred) progeny derived from interspecific crosses and believed that this approach would invariably lead to greater utilization of the favorable attributes hidden in tomato exotics (Rick 1983).

## Abbreviations

QTL: quantitative trait locus
